# Cranial Nerve Noninvasive Neuromodulation in Adults With Neurological Conditions: Protocol for a Scoping Review

**DOI:** 10.2196/29965

**Published:** 2021-07-28

**Authors:** Keaton Boughen, Tyler Neil, Shayan Dullemond, Kevin Lutowicz, Ahmed Bilgasem, Tyler Hastings, Dina Brooks, Julie Vaughan-Graham

**Affiliations:** 1 Physiotherapy Program, School of Rehabilitation Science Faculty of Health Sciences McMaster University Hamilton, ON Canada; 2 Department of Physical Therapy Faculty of Medicine University of Toronto Toronto, ON Canada

**Keywords:** cranial nerve, neurological conditions, neurology, neuromodulation, neurorehabilitation, physical therapy, portable neuromodulation stimulation device, rehabilitation, scoping review, translingual neurostimulation

## Abstract

**Background:**

Cranial nerve noninvasive neuromodulation (CN-NINM) via translingual nerve stimulation (TLNS) is a promising new intervention combined with neurological rehabilitation to improve outcomes for persons with neurological conditions. A portable neuromodulation stimulation (PoNS) device rests on the tongue and stimulates cranial nerves V and VII (trigeminal and facial nerves, respectively). Emerging evidence suggests that CN-NINM using the PoNS device, combined with targeted physical therapy, improves balance and gait outcomes but has not yet been comprehensively reviewed.

**Objective:**

This review will describe CN-NINM via TLNS and its applications, effects, and implications for rehabilitation science in adult populations with neurological conditions. We will identify how CN-NINM via TLNS is currently being incorporated into neurological rehabilitation and identify gaps in evidence with respect to this novel technology.

**Methods:**

Joanna Briggs Institute methodology will be used to conduct this scoping review. Electronic databases MEDLINE, AMED, CINAHL, Embase, and Web of Science will be searched, as well as gray literature databases ProQuest, DuckDuckGo, and Google. Studies published in English and French between 2000 and 2021 will be included. Two reviewers will independently screen all titles and abstracts and full-text papers that meet the inclusion criteria. Data will be extracted and collated in a table to synthesize the results. Extracted data will be reported in a comprehensive summary.

**Results:**

The final manuscript is intended for submission to an indexed journal in September 2021.

**Conclusions:**

This scoping review will be the first, to our knowledge, to address the current evidence on CN-NINM. The results will inform the use of CN-NINM in neurological rehabilitation and the development of recommendations for future research.

**Trial Registration:**

Open Science Framework 10.17605/OSF.IO/XZQFM; https://osf.io/xzqfm

**International Registered Report Identifier (IRRID):**

PRR1-10.2196/29965

## Introduction

### Background

Neurological disorders are currently the leading cause of disability worldwide, and the burden of death and disability caused by them is increasing [1,2]. Stroke, multiple sclerosis (MS), and traumatic brain injury (TBI) are among the largest contributors to disability and mortality worldwide [1,3]. Although the global age-standardized incidence, mortality, and prevalence rates of neurological conditions have been decreasing from 1990 to 2015, years lived with disability and death from neurological conditions has been increasing over the same period [1]. This is consistent with a continually aging population and suggests that people with neurological conditions are living longer with persistent disability [1].

Recent literature has explored the burden of disability among patients with neurological conditions [4-6]. Older adults living with neurological disorders are at a significantly higher risk of falls compared to healthy older adults [4]. A systematic review by Lai et al [4] reported that approximately 73% of individuals who have sustained a stroke will experience a fall in the first 6 months after discharge from hospital. Additionally, motor function deficits make common activities challenging for stroke survivors, such as reaching, grasping, and holding onto household objects [5]. Similar functional impairments have been observed in people with MS. Coote et al [6] reported that over a 6-month period, 71% of older adults with MS sustained a fall compared to 41% of age-matched healthy controls. Individuals with MS who experienced a fall were much more likely to sustain serious injury [6]. As the neurological burden of disease is expected to grow over the coming decades, the development of new, effective rehabilitation interventions to improve the lives of people with neurological conditions is of utmost importance [1].

Cranial nerve noninvasive neuromodulation (CN-NINM) is a novel and emerging technology, grounded in mechanisms that facilitate neuroplasticity, for targeted use in individuals with neurological conditions [7-12]. CN-NINM involves translingual neurostimulation (TLNS) with a portable neuromodulation stimulation (PoNS) device [13-16]. Use of the PoNS device, combined with targeted physical therapy, aims to address longstanding balance and gait deficits in people with neurological conditions [13,14,16].

The PoNS device is equipped with an electrode array that is placed on the anterior dorsal surface of the individual’s tongue [10,13,17]. Electrical impulses are delivered via the PoNS device to the tongue, which stimulate the trigeminal (CN V) and the facial (CN VII) nerves [16,18-20]. Other CNs have been reported to be stimulated as well, including the glossopharyngeal (CN IX), vagal (CN X), and hypoglossal (CN XII) nerves [11,18]. Stimulation of the CNs subsequently leads to the stimulation of targeted areas in the brainstem and cerebellum through the lingual branch of CN V and chorda tympani branch of CN VII [18]. Repetitive stimulation of structures in the brainstem and cerebellum potentiates a cascade of central nervous system neuromodulation [21,22]. Combined with physical therapy, this procedure potentiates significant long-term neuroplastic change [10].

There are a number of advantages to TLNS using the PoNS device for neurological rehabilitation. First, it is a portable noninvasive therapy amenable to home use and can enable higher-frequency and -intensity rehabilitation programs [13,15,17]. Second, to date, there have been no reports of serious adverse events with the use of the PoNS device [23]. Third, this procedure has broad applicability to a wide variety of neurological conditions [23]. Finally, CN-NINM has a rapid effect; neuroplastic changes have been demonstrated in several brain regions after only 5 days of use [11].

The PoNS device uses a biphasic waveform designed to ensure net 0 direct current to minimize tissue irritation [16,23-25]. Voltage and timing are preprogrammed and cannot be adjusted by the user, but the user can adjust the intensity by changing the pulse width [24,25]. There are a wide range of parameters (pulse width, frequency, and intensity) used in the literature; however, optimal parameters remain to be established [7,25]. Intensity of the intervention is variable, ranging 1-3 times per day, while treatment duration ranges between 2 weeks to 7 months [25-33]. Treatment sessions range 20-90 minutes and are typically combined with an active physical rehabilitation program [25,29,31,32]. Two frequencies have been reported in the literature, a high-frequency pulse (150 pulses/second) and a low-frequency pulse (0.08 pulses/second) [32]. Low-frequency pulses, however, are only delivered by the low stimulation pulse device that has been developed exclusively for research purposes (personal communication from Helius Medical Technologies, May 2021).

Case studies and case series have elucidated promising initial clinical applications of CN-NINM via TLNS in a variety of neurological conditions. For example, significant improvements in chronic balance deficits, reducing falls, and improving gait have been reported in individuals living with moderate TBI [7,8,14,15]. Improvements have also been demonstrated in mobility, incidence of falls, and balance in individuals living with stroke [15,27-29]. Lastly, CN-NINM has demonstrated improvements in gait, balance, posture, and self-observed disability in individuals with balance disorders [7].

A preliminary search of PROSPERO, MEDLINE, the Cochrane Database of Systematic Reviews, and Joanna Briggs Institute (JBI) Evidence Synthesis was conducted on February 14, 2021. In the preliminary search, no current or ongoing scoping reviews were identified. Papa et al [11] identified a systematic review that assessed the efficacy of CN-NINM for nervous system disorders [11]. A recent narrative review by Diep et al [7] summarized and appraised the available evidence for TLNS. However, considering the nature of narrative reviews, there is an inherent high risk of bias [34]. This scoping review of CN-NINM will expand on these previous reviews by systematically searching both peer-reviewed and gray literature. Here we performed a scoping review because it involves the analysis, synthesis, and reinterpretation of a broad range of evidence, including different study designs and nonresearch articles, to provide clarity on the emerging field of CN-NINM [35,36]. This will enable clinicians and researchers to gain an understanding of the current state of knowledge with respect to CN-NINM.

### Objectives

The objectives of this scoping review are to assess and understand the evidence available for CN-NINM, including a description of CN-NINM and its mechanism of action, the devices utilized, and for which conditions or clinical symptoms evidence is currently available. Furthermore, we will explore the effects of CN-NINM outlined by previous research efforts, and how CN-NINM is being incorporated into neurological rehabilitation. The current gaps in the literature will be identified, and the implications for rehabilitation science and future research directions will be discussed. This will be the first scoping review of the CN-NINM evidence base; therefore, it will be essential for informing future research and health care policies. The objectives, inclusion criteria, and methods for this scoping review were specified in advance and documented on Open Sciences Framework registries [37]. 

### Review Questions

The following questions have been addressed in this review:

1. What is CN-NINM, and how is CN-NINM being delivered?
2. What are typical parameters used in CN-NINM?
3. Which conditions or clinical symptoms have evidence supporting the use of CN-NINM?
4. How is CN-NINM being incorporated into neurological rehabilitation?
5. What has previous research shown on the effects of CN-NINM in neurorehabilitation?
6. What are the current gaps in the evidence base and the implications for rehabilitation science?

## Methods

### Study Design and Ethics Approval

The proposed scoping review will follow the JBI methodology for scoping reviews [38]. The study is designed and will be conducted in accordance with the PRISMA-ScR (Preferred Reporting Items for Systematic Reviews and Meta-Analyses extension for Scoping Reviews) framework [39]. The objectives, inclusion criteria, and methods for this scoping review were specified in advance and documented on Open Sciences Framework registries [37].

### Inclusion and Exclusion Criteria

Eligibility criteria were established a priori using the population, concept, context framework. Studies will be selected in accordance with the following criteria.

#### Population

This review will consider studies that involve adults with neurological conditions (≥18 years of age) including but not limited to MS, mild TBI including concussions, moderate TBI, severe TBI, and stroke.

#### Concept

The concepts to be explored include CN-NINM, PoNS, and TLNS. We will exclude other forms of invasive and noninvasive brain stimulation such as functional electrical stimulation, transcranial magnetic stimulation, deep-brain stimulation, and transcranial electrical stimulation techniques.

#### Context

This review will consider studies conducted in any context and geographical location to widely explore its application in neurological rehabilitation.

#### Types of Sources

In lieu of the exploratory nature of this scoping review, no restrictions will be placed on the types of sources included in this review. This scoping review will consider including studies with quantitative, qualitative, and mixed methods designs. Furthermore, systematic reviews and gray literature including nonresearch articles, opinion papers, texts, as well as documents from relevant websites will be considered. Studies in English and French published since 2000 will be included.

### Search Strategy

The search strategy will identify peer-reviewed articles and gray literature to explore the full breadth and depth of available evidence. An initial limited search of MEDLINE and CINAHL databases was undertaken to identify articles on the topic. The text words contained in the titles and abstracts of relevant articles were used to develop a full search strategy for MEDLINE (Multimedia Appendix 1). An academic librarian was consulted for suggestions regarding key concepts and Medical Subject Headings. The academic librarian approved the search strategy for MEDLINE, which will be adapted for the other respective databases. Boolean operators (ie, “OR” and “AND”) will be used to combine and refine search terms and concepts. The search will be iterative in nature; therefore, additional search terms may be identified and incorporated into the search strategy. The review team will hand-search all reference lists of the included articles to identify additional studies of relevance.

Articles in English and French, published from January 2000 to the present will be included. This time frame is deemed appropriate as CN-NINM is a novel neuromodulation technique that emerged from the sensory substitution research performed in the early 1990s at the Tactile Communication and Neurorehabilitation Laboratory at the University of Wisconsin-Madison [16,40].

The databases to be searched include MEDLINE, AMED, CINAHL, Embase, and Web of Science. Sources of unpublished studies and gray literature will include ProQuest, DuckDuckGo, and Google. Gray literature will also be targeted from relevant health or scientific organizations pertinent to CN-NINM (eg, Helius Medical Technologies Inc).

For the gray literature search strategy, consistent terms will be used for each browser. The date and time as well as the results of the search will be documented. The first 5 pages of results will be screened. If a document meets the inclusion criteria, a backward and forward reference search will be performed to identify other relevant documents.

### Selection Process

After the search, all identified records will be collated and imported to Zotero reference management software (Center for History and New Media, George Mason University) and duplicates will be removed. The research team will screen the first 10 citations of the initial MEDLINE search to assess the inclusion and exclusion criteria and researcher agreement. Any discrepancies will be discussed and resolved collectively among the research team. The titles and abstracts of the remaining articles will be screened by groups of 2 researchers for assessment against the inclusion criteria identified a priori. Screening differences will be resolved between the 2 researchers, and in cases where an agreement cannot be reached, senior researchers will be consulted. Researchers will categorize studies as “include” or “exclude” to identify articles for full-text screening. Potentially relevant papers will be retrieved in full, and their citation details imported into Covidence screening and data extraction tool (Veritas Health Innovation). Full-text review will be undertaken by groups of 2 researchers. Any disagreements between researchers with regard to the inclusion and exclusion criteria will be resolved through discussion and debate; if a consensus is not reached, a senior researcher will be consulted. Reasons for exclusion of full-text papers that do not meet the inclusion criteria will be recorded and reported in the scoping review.

Articles published in French will undergo 2-stage screening by a senior researcher and 1 author. Any disagreements during the full-text review will be resolved through discussion with the entire research team.

The results of the search will be reported in full and presented in a PRISMA-ScR flow diagram [39].

### Data Extraction

Data extraction will be completed by groups of 2 researchers by using a data extraction tool developed by the research team. To ensure accurate data collection, the extracted data will be compared; discrepancies will be resolved through consensus, or a third researcher will serve as an arbitrator. The data extracted will include specific details about the study population, concept, context, study methods, and key findings relevant to the research questions. Authors of those articles will be contacted to request missing or additional data, where required.

A draft extraction table is provided in Multimedia Appendix 2 and includes minor revisions to the original JBI template [38]. Revisions include examples of details or results extracted from studies to align with the objective of this scoping review. The research team will trial the data extraction table on 2 or 3 sources to ensure that all relevant results are extracted [38]. Modifications will be identified in the full scoping review report.

### Data Presentation

The collected data will be presented in a tabular or graphical format that aligns with the scoping review’s proposed research questions. A summary and synthesis of the findings and discussion of the review will accompany the tabulated or charted data. The full scoping review will be reported in accordance with the PRISMA-ScR checklist [39].

## Results

The collated results will be presented in a scoping review publication. The research team has already initiated study activities, and the results will be available by September 2021.

## Discussion

### Overview

People living with neurological conditions are living longer, often with persistent disabilities that have become refractory to rehabilitation [1]. CN-NINM is a promising new intervention designed to augment physical rehabilitation by harnessing neuroplastic mechanisms to ameliorate persistent movement disabilities related to neurological conditions [1]. It is important for clinicians and researchers to understand the current state of knowledge with respect to CN-NINM, and a scoping review allows for comprehensive exploration of the available literature. This will be, to our knowledge, the first scoping review to comprehensively map the available literature on the use of CN-NINM in neurological rehabilitation. Mapping the literature will yield an outline of current studies and resources within the field, identify current gaps in the evidence base, and provide recommendations for future research. Furthermore, the review will explore how CN-NINM is being incorporated into neurological rehabilitation and its effects, with implications for both researchers and clinicians.

A systematic review by Papa et al [11] reported numerous studies that used the PoNS device and investigated its effects on balance and sensory-motor coordination in a variety of central and peripheral nervous system conditions. Papa et al [11] highlighted the advantages of the device, its noninvasive nature and portability, and its effects on several brain regions. Similarly, the narrative review by Diep et al [7] acknowledges that CN-NINM can be feasibly and safely administered to people with diverse neurological conditions. CN-NINM was found to improve balance and gait symptoms secondary to chronic mild-to-moderate TBI, balance disorders, spinal cord injuries, and Parkinson disease [7]. Both Papa et al [11] and Diep et al [7] highlighted the need for further studies to examine the use, applications, and effects of CN-NINM. As such, our rigorous scoping review will expand on these previous, but different, review methods to provide clarity on the breadth of the available evidence. Furthermore, it will identify research gaps by specifically exploring typical parameters used (pulse-width, frequency, and intensity), length of rehabilitation programs, and whether CN-NINM is typically incorporated as an adjunct intervention within neurological rehabilitation programs.

### Limitations

The limitations of this scoping review include the following. First, the search was limited to 5 databases and 3 gray literature sources; hence, it is possible that pertinent literature may have been missed. However, these databases were chosen on the recommendation of an academic librarian at the McMaster University Health Sciences Library and represent the most extensive health science databases. Second, the gray literature search results may not be reproducible owing to the date, time, and location of its conductance. Third, the articles screened and selected will be restricted to those published in English and French, which is an inherent limitation as pertinent literature on the topic may be published in other languages, thus being potentially missed in our search.

### Conclusions

This scoping review will provide a comprehensive analysis, synthesis, and interpretation of the available literature on the use of CN-NINM in adults with neurological conditions. It will provide an up-to-date evidence summary for neurological rehabilitation specialists and will identify research gaps and limitations in the current literature. This knowledge is essential to guide future studies on CN-NINM. 

## Appendix

Multimedia Appendix 1 MEDLINE search conducted on February 19, 2021.

Multimedia Appendix 2 Data extraction instrument.

## Abbreviations

CN-NINM: cranial nerve noninvasive neuromodulation
JBI: Joanna Briggs Institute
MS: multiple sclerosis
PoNS: portable neuromodulation stimulator
PRIMSA-ScR: Preferred Reporting Items for Systematic Reviews and Meta-Analyses extension for Scoping Reviews
TBI: traumatic brain injury
TLNS: translingual nerve stimulation

## References

[1] GBD 2016 Neurology Collaborators (2019). Global, regional, and national burden of neurological disorders, 1990-2016: a systematic analysis for the Global Burden of Disease Study 2016. Lancet Neurol.

[2] Thakur KT, Albanese E, Giannakopoulos P, Jette N, Linde M, Prince MJ, Steiner TJ, Dua T, Patel V, Chisholm D, Dua T, Laxminarayan R, Medina-Mora ML (2016). Neurological Disorders. Disease Control Priorities, Third Edition (volume 4): Mental, Neurological, and Substance Use Disorders.

[3] Stovner LJ, Hoff JM, Svalheim S, Gilhus NE (2014). Neurological disorders in the Global Burden of Disease 2010 study. Acta Neurol Scand Suppl.

[4] Lai C, Chen H, Liou T, Li W, Chen S (2019). Exercise Interventions for Individuals With Neurological Disorders: A Systematic Review of Systematic Reviews. Am J Phys Med Rehabil.

[5] Hatem S, Saussez G, Della Faille M, Prist V, Zhang X, Dispa D, Bleyenheuft Y (2016). Rehabilitation of Motor Function after Stroke: A Multiple Systematic Review Focused on Techniques to Stimulate Upper Extremity Recovery. Front Hum Neurosci.

[6] Coote S, Comber L, Quinn G, Santoyo-Medina C, Kalron A, Gunn H (2020). Falls in People with Multiple Sclerosis: Risk Identification, Intervention, and Future Directions. Int J MS Care.

[7] Diep D, Lam A, Ko G (2020). A Review of the Evidence and Current Applications of Portable Translingual Neurostimulation Technology. Neuromodulation.

[8] Fickling SD, Greene T, Greene D, Frehlick Z, Campbell N, Etheridge T, Smith CJ, Bollinger F, Danilov Y, Rizzotti R, Livingstone AC, Lakhani B, D'Arcy RCN (2020). Brain Vital Signs Detect Cognitive Improvements During Combined Physical Therapy and Neuromodulation in Rehabilitation From Severe Traumatic Brain Injury: A Case Report. Front Hum Neurosci.

[9] Frehlick Z, Lakhani B, Fickling SD, Livingstone AC, Danilov Y, Sackier JM, D'Arcy RCN (2019). Human translingual neurostimulation alters resting brain activity in high-density EEG. J Neuroeng Rehabil.

[10] Kaczmarek K (2011). The tongue display unit (TDU) for electrotactile spatiotemporal pattern presentation. Sci Iran D Comput Sci Eng Electr Eng.

[11] Papa L, LaMee A, Tan CN, Hill-Pryor C (2014). Systematic review and meta-analysis of noninvasive cranial nerve neuromodulation for nervous system disorders. Arch Phys Med Rehabil.

[12] Wildenberg JC, Tyler ME, Danilov YP, Kaczmarek KA, Meyerand ME (2013). Altered connectivity of the balance processing network after tongue stimulation in balance-impaired individuals. Brain Connect.

[13] Galea M, Cofré Lizama LE, Bastani A, Panisset M, Khan F (2017). Cranial nerve non-invasive neuromodulation improves gait and balance in stroke survivors: A pilot randomised controlled trial. Brain Stimul.

[14] Wardini R, Moses M (2017). Portable neuromodulation stimulation (PoNSTM) therapy efficacy for the treatment of traumatic brain injury compared to standard of care. Brain Inj.

[15] Danilov Y, Skinner K, Subbotin A, Verbny Y, Tyler M, Kaczmarek K (2013). Effects of CN-NINM intervention on chronic stroke rehabilitation: a case study.

[16] Danilov Y, Tyler M, Kaczmarek K, Skinner K (2014). New approach to neurorehabilitation: cranial nerve noninvasive neuromodulation (CN-NINM) technology.

[17] Leonard G, Lapierre Y, Chen J, Wardini R, Crane J, Ptito A (2017). Noninvasive tongue stimulation combined with intensive cognitive and physical rehabilitation induces neuroplastic changes in patients with multiple sclerosis: A multimodal neuroimaging study. Mult Scler J Exp Transl Clin.

[18] Danilov Y (2017). Translingual Neurostimulation (TLNS): A Novel Approach to Neurorehabilitation. Phys Med Rehabil Int.

[19] Bastani A, Cofré Lizama LE, Zoghi M, Blashki G, Davis S, Kaye AH, Khan F, Galea MP (2018). The combined effect of cranial-nerve non-invasive neuromodulation with high-intensity physiotherapy on gait and balance in a patient with cerebellar degeneration: a case report. Cerebellum Ataxias.

[20] Cofré Lizama LE, Bastani A, Panisset MG, Drummond K, Khan F, Galea MP (2018). A novel neuromodulation technique for the rehabilitation of balance and gait: A case study. J Clin Neurosci.

[21] Hou J, Kulkarni A, Tellapragada N, Nair V, Danilov Y, Kaczmarek K, Meyerand B, Tyler M, Prabhakaran V (2020). Translingual Neural Stimulation With the Portable Neuromodulation Stimulator (PoNS®) Induces Structural Changes Leading to Functional Recovery In Patients With Mild-To-Moderate Traumatic Brain Injury. EMJ Radiol.

[22] Chisholm AE, Malik R, Blouin J, Borisoff J, Forwell S, Lam T (2014). Feasibility of sensory tongue stimulation combined with task-specific therapy in people with spinal cord injury: a case study. J Neuroeng Rehabil.

[23] Tyler M, Skinner K, Prabhakaran V, Kaczmarek K, Danilov Y (2019). Translingual Neurostimulation for the Treatment of Chronic Symptoms Due to Mild-to-Moderate Traumatic Brain Injury. Arch Rehabil Res Clin Transl.

[24] Danilov Y, Tyler M, Kazcmarek K (2012). New approach to chronic TBI rehabilitation: cranial nerve noninvasive neuromodulation (CN-NINM technology).

[25] Tyler ME, Kaczmarek KA, Rust KL, Subbotin AM, Skinner KL, Danilov YP (2014). Non-invasive neuromodulation to improve gait in chronic multiple sclerosis: a randomized double blind controlled pilot trial. J Neuroeng Rehabil.

[26] D'Arcy RCN, Greene T, Greene D, Frehlick Z, Fickling SD, Campbell N, Etheridge T, Smith C, Bollinger F, Danilov Y, Livingstone A, Tannouri P, Martin P, Lakhani B (2020). Portable neuromodulation induces neuroplasticity to re-activate motor function recovery from brain injury: a high-density MEG case study. J Neuroeng Rehabil.

[27] Paltin D, Danilov Y, Tyler M (2018). Direct and indirect benefits of translingual neurostimulation technology for neurorehabilitation of chronic stroke symptoms. Brain-machine interfaces: uses and developments.

[28] Liegl K (2013). Potential Benefits and Withdrawal Effects of Cranial Nerve Non-invasive Neuromodulation on Functional Mobility for Individuals with Traumatic Brain Injury. University of Wisconsin Milwaukee.

[29] Paltin D, Tyler M, Danilov Y (2017). Cognitive enhancement exciting discovery using trans-lingual neuro-stimulation. J Neurol Neurorehabil Res.

[30] Paltin D, Tyler M, Danilov Y (2017). CN-NINM Intervention For The Neurorehabilitation Of Disordered Speech And Emotion. Med Sci Sports Exerc.

[31] Wildenberg JC, Tyler ME, Danilov YP, Kaczmarek KA, Meyerand ME (2011). High-resolution fMRI detects neuromodulation of individual brainstem nuclei by electrical tongue stimulation in balance-impaired individuals. Neuroimage.

[32] Ptito A, Papa L, Gregory K, Folmer RL, Walker WC, Prabhakaran V, Wardini R, Skinner K, Yochelson M (2020). A Prospective, Multicenter Study to Assess the Safety and Efficacy of Translingual Neurostimulation Plus Physical Therapy for the Treatment of a Chronic Balance Deficit Due to Mild-to-Moderate Traumatic Brain Injury. Neuromodulation.

[33] Danilov Y, Tyler M, Kaczmarek K (2011). Rehabilitation of multiple sclerosis and Parkinson?s symptoms using cranial nerve non-invasive neuromodulation (CN-NINM). Mov Disord.

[34] Peters M, Godfrey C, McInerney P, Munn Z, Tricco A, Khalil H (2020). JBI Systematic Reviews. JBI Manual for Evidence Synthesis.

[35] Peters M, Godfrey C, McInerney P, Munn Z, Tricco A, Khalil H (2020). Scoping Reviews. JBI Manual for Evidence Synthesis.

[36] Arksey H, O'Malley L (2005). Scoping studies: towards a methodological framework. Int J Soc Res Methodol.

[37] Boughen K, Dullemond S, Neil T, Bilgasem A, Lutowicz K, Hastings T, Brooks D, Vaughn-Graham J (2021). Cranial nerve non-invasive neuromodulation in adults with neurological conditions: a scoping review protocol 2021. Open Sciences Framework.

[38] Peters M, Godfrey C, McInerney P, Munn Z, Tricco A, Khalil H (2015). Methodology for JBI Scoping Reviews. The Joanna Briggs Institute Reviewers' Manual 2015.

[39] Tricco A, Lillie E, Zarin W, O'Brien KK, Colquhoun H, Levac D, Moher D, Peters MDJ, Horsley T, Weeks L, Hempel S, Akl EA, Chang C, McGowan J, Stewart L, Hartling L, Aldcroft A, Wilson MG, Garritty C, Lewin S, Godfrey CM, Macdonald MT, Langlois EV, Soares-Weiser KJ, Moriarty J, Clifford T, Tunçalp Ö, Straus SE (2018). PRISMA Extension for Scoping Reviews (PRISMA-ScR): Checklist and Explanation. Ann Intern Med.

[40] About Helius. Helius Medical Technologies.

