# Supplementary material for: Cranial Nerve Noninvasive Neuromodulation in Adults With Neurological Conditions: Protocol for a Scoping Review
Source: JMIR Res Protoc. 2021 Jul 28;10(7):e29965. doi: 10.2196/29965 (PMC8367107; doi:10.2196/29965)
Supplement: Multimedia Appendix 1 [file resprot_v10i7e29965_app1.docx]

## Multimedia Appendix 1

MEDLINE search conducted on February 19, 2021

| **Search** | **Query** | **Records retrieved** |
| --- | --- | --- |
| #1 | exp Central Nervous System Diseases/ OR exp Brain Injuries/ OR exp Multiple Sclerosis/ OR Headache/ OR exp Headache Disorders/ OR "Central Nervous System Disorders".ti,ab. OR ((brain or head) adj2 injur*).ti,ab. OR multiple sclerosis.ti,ab. OR headache.ti,ab. OR ((head or cranial) adj2 pain).ti,ab. OR Stroke.ti,ab. OR concuss*.ti,ab. OR mtbi.ti,ab. | 1732226 |
| #2 | exp Cranial Nerves/ OR ((cranial or trigeminal or vagus or "lingual branch" or tongue or translingual or facial) adj3 nerv*).ti,ab. | 140389 |
|  | exp Electric Stimulation Therapy/ OR ("Portable translingual neurostimulation" or "TLNS" or "HELIUS" or "Pons" or "CN-NINM" or "Neuromodulation" or "Electrodes" or "Stimulat*" or "Neurostimulat*").ti,ab. | 1289223 |
| #3 | #1 AND #2 AND #3 | 2779 |
| Limit: 2000-present | | 2108 |
