# Supplementary material for: Cranial Nerve Noninvasive Neuromodulation in Adults With Neurological Conditions: Protocol for a Scoping Review
Source: JMIR Res Protoc. 2021 Jul 28;10(7):e29965. doi: 10.2196/29965 (PMC8367107; doi:10.2196/29965)
Supplement: Multimedia Appendix 2 [file resprot_v10i7e29965_app2.docx]

## Multimedia Appendix 2

Data extraction instrument

| **Scoping Review details** | |
| --- | --- |
| Scoping Review title: |  |
| Review objective/s: |  |
| Review question/s |  |
| **Inclusion/Exclusion Criteria** | |
| Population |  |
| Concept |  |
| Context |  |
| Types of study |  |
| **Evidence source details and characteristics** | |
| Study citation details (e.g. author/s, date, title, journal, volume, issue, pages) |  |
| Country |  |
| Context |  |
| Participants (details e.g. age/sex and number) |  |
| **Details/Results extracted from source of evidence**(in relation to the concept of the scoping review) | |
| Description of what CN-NINM is |  |
| Details on how CN-NINM is being delivered |  |
| Conditions or clinical symptoms that have evidence supporting the use of CN-NINM |  |
| How CN-NINM is being incorporated into neurological rehabilitation |  |
| Details on the effectiveness of CN-NINM |  |
| Current gaps in the evidence base |  |
| Implications for rehabilitation science |  |
| Common parameters used for CN-NINM |  |
| CN-NINM administered alone or in combination with rehabilitation program |  |

## 
